# Right Cervical Vagotomy Aggravates Viral Myocarditis in Mice Via the Cholinergic Anti-inflammatory Pathway

**DOI:** 10.3389/fphar.2017.00025

**Published:** 2017-01-31

**Authors:** Ge Li-Sha, Chen Xing-Xing, Wu Lian-Pin, Zhou De-Pu, Li Xiao-Wei, Lin Jia-Feng, Li Yue-Chun

**Affiliations:** ^1^Department of Pediatrics, Second Affiliated Hospital of Wenzhou Medical UniversityWenzhou, China; ^2^Department of Cardiology, Second Affiliated Hospital of Wenzhou Medical UniversityWenzhou, China; ^3^Department of Cardiology, First Affiliated Hospital of Wenzhou Medical UniversityWenzhou, China

**Keywords:** viral myocarditis, cervical vagotomy, vagal nerve, cholinergic anti-inflammatory pathway, inflammatory cytokines

## Abstract

The autonomic nervous system dysfunction with increased sympathetic activity and withdrawal of vagal activity may play an important role in the pathogenesis of viral myocarditis. The vagus nerve can modulate the immune response and control inflammation through a ‘cholinergic anti-inflammatory pathway’ dependent on the α7-nicotinic acetylcholine receptor (α7nAChR). Although the role of β-adrenergic stimulation on viral myocarditis has been investigated in our pervious studies, the direct effect of vagal tone in this setting has not been yet studied. Therefore, in the present study, we investigated the effects of cervical vagotomy in a murine model of viral myocarditis. In a coxsackievirus B3 murine myocarditis model (Balb/c), effects of right cervical vagotomy and nAChR agonist nicotine on echocardiography, myocardial histopathology, viral RNA, and proinflammatory cytokine levels were studied. We found that right cervical vagotomy inhibited the cholinergic anti-inflammatory pathway, aggravated myocardial lesions, up-regulated the expression of TNF-α, IL-1β, and IL-6, and worsened the impaired left ventricular function in murine viral myocarditis, and these changes were reversed by co-treatment with nicotine by activating the cholinergic anti-inflammatory pathway. These results indicate that vagal nerve plays an important role in mediating the anti-inflammatory effect in viral myocarditis, and that cholinergic stimulation with nicotine also plays its peripheral anti-inflammatory role relying on α7nAChR, without requirement for the integrity of vagal nerve in the model. The findings suggest that vagus nerve stimulation mediated inhibition of the inflammatory processes likely provide important benefits in myocarditis treatment.

## Introduction

Viral myocarditis is an inflammatory disease involving myocardial cells and the myocardial interstitium. The clinical manifestations of viral myocarditis vary; mild cases may simply have symptoms such as palpitations and fatigue, while severe cases may involve cardiogenic shock, syncope and even sudden death. The majority of viral myocarditis cases recover after treatment, while a few patients eventually develop dilated cardiomyopathy due to ongoing injury with persistent viral infection or immune response (Cooper, 2009). The incidence of viral myocarditis in adults and adolescents increases year by year (Cooper, 2009). Coxsackie virus and other picornaviruses lead to more than 50% of cases of acute myocarditis and approximately 25% of cases of dilated cardiomyopathy. Epidemiological data shows that myocarditis is the main cause of heart failure in patients less than 40 years of age (Yajima and Knowlton, 2009).

The development of viral myocarditis is divided into three stages (Dennert et al., 2008). In the first stage, viral infection causes direct myocardial damage and simultaneously activates the innate immune system to help clear the pathogens. The second stage involves abnormal immune regulation secondary to cardiomyocyte injury in the first phase. This immune response is different from that of the first stage, and it is the main cause of the progression of myocarditis, which is harmful to the body. Finally, extensive myocardial damage leads to the formation of dilated cardiomyopathy. At present, there is still no effective antiviral therapy for viral myocarditis, except for symptomatic treatment (Henke et al., 2001; Liu et al., 2005; Magnani and Dec, 2006; Li, 2016). Despite no effective antiviral therapy, but inflammatory cytokine-associated damage to myocytes has been shown to be involved (Cooper, 2009; Yajima and Knowlton, 2009; Kong et al., 2013). Among them, tumor necrosis factor (TNF)-α, interleukin (IL)-1, and IL-6 are the three representative proinflammatory cytokines of human and animal viral myocarditis. Studies have shown that the overwhelming production of pro-inflammatory cytokines (such as TNF-α, IL-1, and IL-6) significantly aggravates myocarditis while inhibiting the expression of the inflammatory cytokines by gene knockout, significantly improving myocardial injury. Autonomic nervous system dysfunction, characterized by sympathetic activation and vagal withdrawal, is an important contributor to the progression of diverse cardiovascular diseases (Zhang et al., 2009). We have found that excessive sympathetic activation promotes inflammatory immune responses and aggravates inflammatory lesion in viral myocarditis, and the nonselective β-blocker carvedilol was beneficial due to its effects to limit the production of pro-inflammatory cytokines (Li et al., 2008, 2010, 2012a,b; Zheng et al., 2016). These findings suggest that the autonomic nervous system can modulate the immune response and regulate inflammation, and the autonomic nervous system dysfunction may play an important role in viral myocarditis (Li, 2016; Zheng et al., 2016). Until now, however, the role of the vagus nerve stimulation in viral myocarditis has been unclear.

Recent studies indicate that the vagus nerve (which is the longest of the cranial nerves and innervates most of the peripheral organs) can modulate the immune response and control inflammation through a “cholinergic anti-inflammatory pathway” dependent on the α7-nicotinic acetylcholine receptor (α7nAChR) (Borovikova et al., 2000). The cholinergic anti-inflammatory pathway mainly contains the efferent vagal nerve, acetylcholine (ACh) and α7nAchR. The vagus nerve plays a vital role in both signal transmission and output. ACh, the endogenous vagal neurotransmitter, binds with α7nAChR to activate or inhibit the signaling pathway that regulate expression of inflammatory cytokines (Marrero and Bencherif, 2009). The α7nAChR expressed in immune cells and some non-immune cells (Dvorakova et al., 2005) is considered to be an anti-inflammatory target. The anti-inflammatory effect of the α7nAChR has been demonstrated in many experimental models of inflammatory disorders (Guarini et al., 2003; Altavilla et al., 2006; van Westerloo et al., 2006; Huston et al., 2007; Pavlov et al., 2007; Leib et al., 2011; Lee, 2013; Park et al., 2013; Safieh-Garabedian et al., 2013; Sun et al., 2013; Zhao et al., 2013). Recently, we have also demonstrated that cholinergic stimulation with α7nAChR agonist nicotine had a protective effects in murine viral myocarditis, and selective α7nAchR antagonist methyllycaconitine had a deleterious effects in the same setting (Zheng et al., 2014; Ge et al., 2015, 2016). Although the pharmacological stimulation or inhibition of the cholinergic anti-inflammatory pathway has been studied, the direct effect of vagal nerve in the viral myocarditis model, however, was not clearly investigated. Therefore, in the present study, we further investigated the effects of cervical vagotomy in a murine model of viral myocarditis induced by coxsackievirus B3 (CVB3) infection.

## Materials and Methods

### Ethics Statement

The study was in line with China Animal Welfare Law and approved by the Animal Ethics Committee of Wenzhou Medical University. Experimental mice were injected intraperitoneally with 2% pentobarbital sodium (40 mg/kg) before surgery. All of the operations were performed under anesthesia. All experimental animals were sacrificed with an overdose of pentobarbital (100 mg/kg, one dose intraperitoneally).

### Division of Groups and Establishment of Viral Myocarditis Model

Specific pathogen-free inbred, 4-week-old, male Balb/c mice, obtained from Shanghai Laboratory Animal Center, China, were randomly divided into five groups according to different treatments: (1) Control group (*n* = 40), normal saline (1.2 mg/kg/d) only. (2) Sham group (*n* = 40), CVB3 0.1 ml + right cervical vagus nerve dissociation only without mutilation of cervical vagus nerve + normal saline (1.2 mg/kg/d). (3) Nic group (*n* = 40), CVB3 0.1 ml + nicotine (1.2 mg/kg/d). (4) Vag group (*n* = 40), CVB3 0.1 ml + right cervical vagotomy + normal saline (1.2 mg/kg/d). (5) Vag+Nic group (*n* = 40), CVB3 0.1 ml + right cervical vagotomy + nicotine (1.2 mg/kg/d). Mice prepared for vagotomy were fasted for 12 h in advance with free access to drinking water. After intraperitoneal injection of 2% pentobarbital sodium (40 mg/kg), the neck and chest was shaved and sterilized. Then, mice were fixed onto the operating table in the supine position, and a one-centimeter incision was made in the middle of the neck. The tissue was separated layer by layer, the right cervical vagal nerve was freed from the carotid sheath and cut off, and the incision was sutured. The cervical vagal nerve in the sham-operated group was only isolated from surrounding tissue but not transected., and the rest of the procedures were performed in the same way as in the Vag group. All procedures followed the principles of aseptic technique, and repeated doses of pentobarbital sodium were infused as necessary throughout the operation. After all surgeries were complete, all mice except for the control group mice were inoculated intraperitoneally with 1.0 × 10^6^ plaque-forming units (pfu) × of CVB3 (strain Nancy) diluted in phosphate-buffered saline to a final volume of 0.1 ml. Control mice were inoculated intraperitoneally with 0.1 ml normal saline solution in the same manner. The day of virus inoculation was defined as day 0.

### Drug Administration

Nicotine (product number: N3876) was purchased from Sigma-Aldrich Co. Twenty-four hours after virus inoculation, mice were intraperitoneally injected with nicotine (0.4 mg/kg, thrice per day) for 14 continuous days. Simultaneously, the control group, sham-operated group and vagotomy group were intraperitoneally given with equal dose of normal saline. Animals were killed at day 7 and 14, and heart samples were obtained for pathological examination, fluorescent quantitative real-time PCR and enzyme-linked immunosorbent assay (ELISA).

### Echocardiography

Transthoracic echocardiography was performed using a Sonos 5500 ultrasound machine (Phillips, USA) with a 12-MHz phased array transducer on days 7 and 14, as previously described (Li et al., 2010). The transducer was covered with the finger of a surgical latex glove filled with ultrasound transmission gel to provide a standoff of 0.5–0.7 cm. The transducer was used at a depth setting of 2 cm to optimize resolution. After anesthesia, the chest was shaved and mice were placed on a heating pad. Two-dimensional, M-mode, Doppler flow images were obtained in parasternal long-axis view. The left ventricular end-systolic and end-diastolic internal diameters (LVESd, LVEDd) were measured over the course of at least 3 consecutive cardiac cycles. The left ventricular ejection fraction (LVEF) and fractional shortening (FS) were then both calculated.

### Myocardial Histopathology

Heart samples obtained after echocardiography were fixed for 12–24 h in 10% formalin, embedded in paraffin and sectioned. The sections were examined under an optical microscope after staining with hematoxylin and eosin, and scored blindly by two observers, as previously described (Li et al., 2010). The scores assigned to these specific sections were averaged. According to the percentage of cellular infiltration and myocardial necrosis in each heart section, the severity was evaluated as follows: 0 = no lesion; 1+ = <25% of the myocardium that is involved; 2+ = 25% to 50%; 3+ = 50 to 75%; and 4+ = >75%.

### Fluorescent Quantitative Real-Time PCR

Total RNA was extracted with the Trizol method (Invitrogen, Carlsbad, CA, USA) according to the manufacturer’s instructions. 2 μg of RNA was used for cDNA synthesis by reverse transcription. An initial denaturation at 95°C for 5 min, 40 cycles of amplification at 95°C for 10 s, and annealing at 60°C for 10 s were conducted. The control gene used was GAPDH. The mouse-specific forward and reverse primers involved in the PCR reaction are as follows, respectively: control gene GAPDH, F-GTGAAGGTCGGTGTGAACGG and R-TCCTGGAAGATGGTGATGGG; CVB3,F-CGGTACCTTTGTGCGCCTGT and R-CAGGCCGCCAACGCAGCC; IL-1β, F-CAGGATGAGGACATGAGCACC and R-CTCTGCAGACTC AAACTCCAC; IL-6, F-GTGGAAATGAGAAAAGAGTTGTGC and R-GACTCT GGCTTTGTCTTTCTTGTT; TNF-α, F-CCACGCTCTTCTGTCTACTGA and R-AAGGTACAACCCATCGGCTG; IFN-γ, F-CAGAGCAGATTATTCTTTTACC and R-ACCTGTGGGTTGTTGACCTC.

### Enzyme-Linked Immunosorbent Assay for Cytokines

Cardiac tissue samples were weighed and sliced into small pieces on dry ice, then homogenized (100 mg tissue per ml of ice-cold homogenizer buffer) and centrifugated at 10000 rpm for 20 min at 4°C. Lowry method was used to calculate the total protein concentration. The supernatants were stored at -80°C for ELISA analysis after dilution and aliquot. Cytokine levels were measured with various ELISA kits (ExCell Bio Co. Ltd, China) according to the manufacturer’s instructions. The sensitivity of the kit was 4 pg/ml for IL-1β, 7 pg/ml for IL-6, 7 pg/ml for TNF-α, and 4 pg/ml for IFN-γ. Cytokine levels were expressed as pg/mg of heart tissue. The experiment was repeated thrice for each heart.

### Statistical Analysis

All data were expressed as the mean ± SD. The statistical analysis was performed using a one-way analysis of variance (ANOVA), followed by Fisher’s protected least significant difference test. Analysis was performed with SPSS 19.0 statistical software. *P* < 0.05 represented statistical significance.

## Results

### Echocardiographic Findings

**FIGURE 1:**
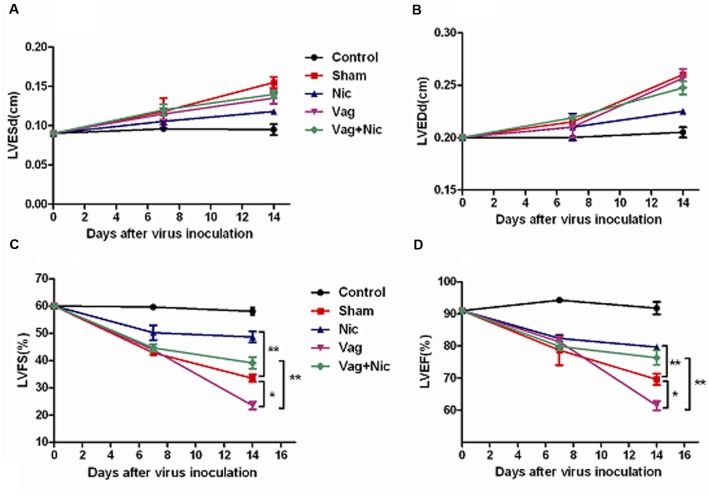
**Echocardiography (*n* = 8 in each group).** **(A)** LVESd = left ventricular end diastolic diameter; **(B)** LVEDd = left ventricular end diastolic diameter; **(C)** LVFS = left ventricular ejection fraction; **(D)** LVEF = left ventricular ejection fraction. ^∗^*P* < 0.05 ^∗∗^*P* < 0.01.

**FIGURE 2:**
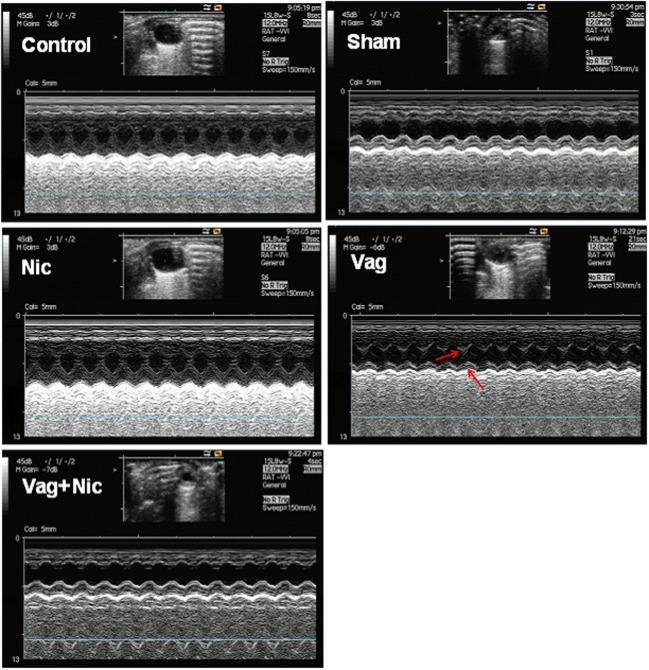
**Representative M-type cardiac ultrasound image on day 14.** It was shown in the figures that the left ventricle movement of Sham, Vag, and Vag + Nic was weaker than that of control and Nic. Arrows indicated that anterior and posterior ventricular wall motion was not synchronized.

The LVEF and FS of the Sham, Nic, Vag, and Vag + Nic groups were significantly decreased compared with the control group on day 7 and 14 (*P* < 0.05). On day 14, LVEF and LVFS of the Nic group were significantly higher than those of the Sham group (*P* < 0.01), and LVEF and LVFS of the Vag group were significantly lower than those of the Sham group (*P* < 0.05). LVEF and LVFS of the Vag + Nic group were significantly higher than those of the Vag group (*P* < 0.01), but no significant differences were found when compared to the Sham group (**Figure 1**). There was no significant difference in the LVESd and LVEDd among the five groups. Representative cardiac ultrasound images are shown in **Figure 2**.

### Myocardial Histopathology

**FIGURE 3:**
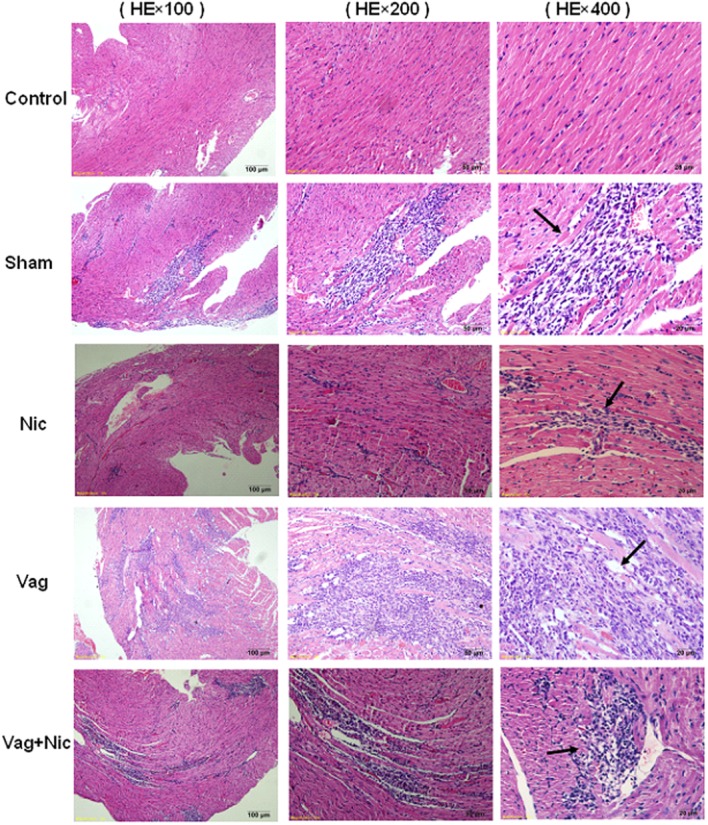
**Representative myocardial pathological changes (Hematoxylin Eosin ×100, ×200, × 400) on day 7**.

On days 7 and 14, no obvious pathological changes in the myocardium of the normal control group were found, and severe injuries were observed in the other four groups of mice infected with CVB3. Under the microscope, myocardial lesions were the most severe in the Vag group, the lightest in the Nic group, and medium in the sham and Vag + Nic groups. The cardiac pathological scores, including cellular infiltration and necrosis, were significantly lower in the Nic group and significantly higher in the Vag group compared with the control group. Pathological scores of the Vag + Nic group were significantly lower than those in the Vag group (**Table 1**). Additionally, the infiltration of inflammatory cells in the myocardium was attenuated, but myocardial necrosis was aggravated on day 14 compared with day 7. Representative hematoxylin-eosin-stained hearts are shown in **Figure 3**.

**Table 1:** Myocardial histologic scores on days 7 and 14.

	*n*	Infiltration		Necrosis	
			
		7 day	14 day	7 day	14 day
Control	8	ND	ND	ND	ND
Sham	8	2.2 ± 0.24	1.6 ± 0.21	1.4 ± 0.10	1.8 ± 0.13
Nic	8	1.3 ± 0.19^∗^	1.0 ± 0.25^∗^	1.0 ± 0.11^∗^	1.3 ± 0.17^∗^
Vag	8	3.4 ± 0.21^∗^	2.3 ± 0.24^∗^	2.1 ± 0.20^∗^	2.5 ± 0.18^∗^
Vag + Nic	8	1.9 ± 0.22^#^	1.5 ± 0.24^#^	1.2 ± 0.15^#^	1.5 ± 0.20^#^


*ND, not detected. ^∗^*P* < 0.05 versus Sham. ^#^*P* < 0.05 versus Vag.*

### Viral RNA Levels

**FIGURE 4:**
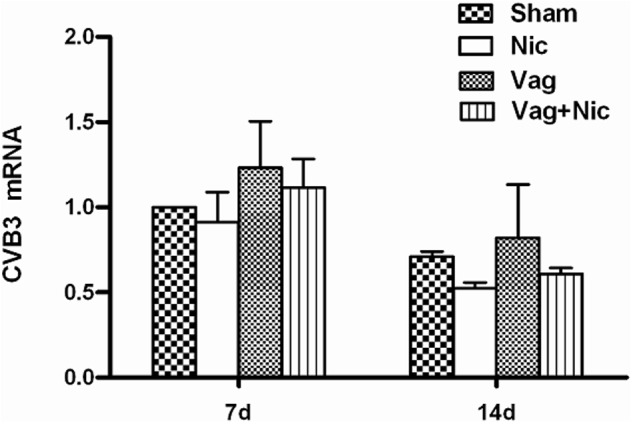
**Expression of CVB3 mRNAs by quantitative PCR analysis in the myocardium of mice on day 7 and 14 (*n* = 8 in each group)**.

Viral abundance was detected in the infected heart tissue on days 7 and 14. The expression of CVB3-RNA was slightly increased in Vag and slightly decreased in Nic compared to Sham, but these effects did not reach statistical significance (**Figure 4**). No significant difference was found in the viral RNA level among all groups.

### Gene Expression of Proinflammatory Cytokines

**FIGURE 5:**
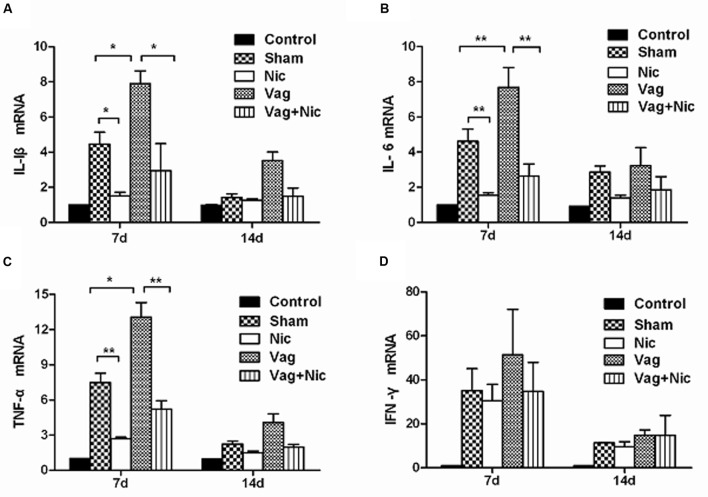
**Expression of cytokine mRNAs by quantitative PCR analysis in the myocardium (*n* = 8 in each group)**. **(A)** IL-1β; **(B)** IL-6; **(C)** TNF-α; **(D)** IFN-γ. ^∗^*P* < 0.05, ^∗∗^*P* < 0.01.

Compared with the Sham group on day 7, the mRNA levels of IL-1β, IL-6, and TNF-α in the myocardium were significantly up-regulated in the Vag group and down-regulated in the Nic group (**Figures 5A–C**). The expression levels of IL-1β, IL-6, and TNF-α in the Vag + Nic group were between the Vag and Nic group and were significantly down-regulated relative to those in the Vag group on day 7. There was no significant difference in the expression of IL-1β, IL-6, and TNF-α in the four myocarditis groups on day 14. We found no significant difference in the expression of IFN-γ in these four groups (**Figure 5D**).

### Protein Expression of Proinflammatory Cytokines

**FIGURE 6:**
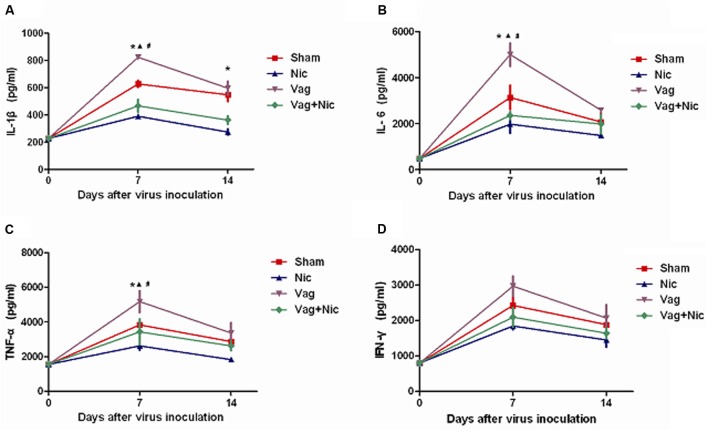
**Cytokine levels measured by ELISA analysis in the myocardium (*n* = 8 in each group).** **(A)** IL-1β; **(B)** IL-6; **(C)** TNF-α; **(D)** IFN-γ. ^∗^*P* < 0.05 Nic versus Sham, 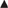 *P* < 0.05 Vag versus Sham, #*P* < 0.05 Vag+ Nic versus Vag.

Compared with the Sham group on day 7, the protein levels of IL-1β, IL-6, and TNF-α in the myocardium were significantly up-regulated in the Vag group and down-regulated in the Nic group (**Figures 6A–C**). The expression levels of IL-1β, IL-6, and TNF-α in the Vag + Nic group were between the Vag and Nic group and were significantly down-regulated relative to those in the Vag group on day 7. The levels of IL-1β, IL-6, and TNF-α in the four myocarditis groups were decreased on day 14, but no significant difference was detected, except for IL-1β. We found no significant difference in the expression of IFN-γ in these four groups (**Figure 6D**).

## Discussion

In the early 21st century, Borovikova et al. (2000) first proposed the concept of the cholinergic anti-inflammatory pathway, a neuroimmunological reflexive signaling pathway with potent anti-inflammatory capacity. Since then, the vagus nerve and its transmitters and receptors have attracted the close attention of researchers. It has been proven that the activation of the cholinergic anti-inflammatory pathway by either pharmacological or electrical stimulation improves various inflammatory diseases and exerts therapeutic effects, and the inhibition of this pathway promotes the inflammatory response and aggravates the disease. Calvillo et al. (2011) indicated that vagus nerve stimulation can reduce the myocardial infarction area, prevent further apoptosis of the damaged cardiomyocytes near the infarction area, lower plasma levels of pro-inflammatory cytokines in ischemia reperfusion rats and inhibit the cholinergic anti-inflammatory pathway with nicotine receptor blockers, further expanding the myocardial infarction area. Similar results have also been reported in cardiovascular diseases such as hypertension (Li et al., 2011) and heart failure (Zhang et al., 2009). Leib et al. (2011) then confirmed that α7nAchR agonist nicotine treatment can relieve the inflammatory injury and myocardial fibrosis in mice with autoimmune myocarditis. Our previous studies (Zheng et al., 2014; Ge et al., 2015, 2016) revealed for the first time that nicotine dose-dependently reduced the severity of viral myocarditis in mice by decreasing the release of proinflammatory cytokines, and acute viral myocarditis is aggravated by treatment with the selective α7nAchR antagonist methyllycaconitine. However, in viral myocarditis, the role of vagus nerve was rarely reported.

This study aimed to further explore the effect of vagus nerve in viral myocarditis. Studies have demonstrated that the protective effects of electrical vagus nerve stimulation on left and right vagus nerve are equivalent, and the anti-inflammatory effect of vagus nerve stimulation does not depend on the nerve’s location (whether on the left or right side) (Bemik et al., 2002). Using right cervical vagotomy and nicotine to inhibit and activate the cholinergic anti-inflammatory pathway, we studied the echocardiography, myocardial histopathology, viral RNA, and proinflammatory cytokine levels in these five groups, and found that vagotomy aggravated myocardial lesions, up-regulated the expression of TNF-α, IL-1β, and IL-6, and worsened the impaired left ventricular function in murine viral myocarditis. On the contrary, nicotine treatment attenuated myocardial lesions, down-regulated the expression of TNF-α, IL-1β, and IL-6, and improved the left cardiac function, which is in consistent with our previous studies (Zheng et al., 2014; Ge et al., 2015, 2016). Moreover, the impaired cardiac function and elevated inflammation in murine viral myocarditis after vagotomy were reversed by co- administration with nicotine through stimulation of the cholinergic anti-inflammatory pathway. These results indicate that right cervical vagotomy aggravates viral myocarditis, and vagal nerve plays an important role in mediating the anti-inflammatory effect in viral myocarditis, and that cholinergic stimulation with nicotine also plays its peripheral anti-inflammatory role and the effects of nicotine do not require the integrity of the vagal nerve in the model. The findings suggest that vagus nerve stimulation mediated inhibition of the inflammatory processes likely provide important benefits. The novel findings extend the understanding of the cholinergic anti-inflammatory pathway efficacy in murine viral myocarditis models (Zheng et al., 2014; Ge et al., 2015, 2016).

The vagus nerve is an important part of the autonomic nervous system, and its nerve endings are widely distributed in the organs of the reticuloendothelial system (Bellinger et al., 2001). In principle, all regions of the heart are innervated by vagus nerves, and in most species, including the human heart, and the supraventricular tissues are more densely innervated than the ventricles (Dhein et al., 2001; Chen et al., 2008). The vagus nerve is a mixed nerve, and the afferent fiber accounts for 80% of its total nerve (Berthoud and Neuhuber, 2000). Previous animal experiments elucidated that afferent vagal fibers play an important role in transferring peripheral inflammatory signals to the central nervous system and efferent vagal activity can significantly regulate both local and systemic inflammation. Vagal activation resulting in cardioprotection is not only associated with heart rate, anti-adrenergic effect, cellular redox states and cell apoptosis, but also related to anti-inflammatory activity (Tsutsumi et al., 2008; Katare et al., 2009; Thayer and Fischer, 2009; Liu et al., 2011). Within this cholinergic anti-inflammatory circut, the vagus nerve functions in both signal integration and signal transmission by evaluating and regulating levels of circulating pro-inflammatory cytokines. Direct electrical stimulation of the distal end of either vagus can reduce serum and tissue levels of proinflammatory cytokines, and vagotomy increases inflammatory injury and makes the animal more vulnerable to the attack of inflammatory stimulation. Borovikova reported that electrical stimulation of the peripheral vagal fibers limited TNF production in the liver of endotoxemia rats and vagotomy enhanced the inflammation and mortality of experimental animals (Borovikova et al., 2000). Ustinova also found that the vagus nerve stimulation can decrease the level of oxyradicals and alleviate the myocardial injury caused by myocardial anaerobic glycolysis and lactic acid accumulation during myocardial ischemia reperfusion (Ustinova and Schultz, 1994). In a bacterial peritonitis model, cutting off the unilateral cervical (van Westerloo et al., 2005) or hypodiaphragmatic vagus nerve (Kessler et al., 2006) elevated plasma IL-1, IL-6, and TNF-α levels, increased the infiltration of peritoneal neutrophil granulocytes and macrophages, and promoted animal mortality. In addtion, vagotomy may influence the recruitment and activity of immune cell to participate in inflammation. Mihaylova and his colleagues found vagotomy strongly decline immune cell counts (including CD4^+^ T cells and CD8^+^ T cells) in the septic spleen (Mihaylova et al., 2014). The previous experiments elucidated that efferent vagal activity can significantly regulate both local and systemic inflammation. In our model, the diminished vagal activity induced by unilateral cervical vagotomy reduced the release of endogenous acetylcholine, with a decrease in the binding of α7nAChR, thereby failing to inhibit inflammation and increasing pro-inflammatory cytokines. The results of the present study are consistent with previous studies (Ustinova and Schultz, 1994; Borovikova et al., 2000; van Westerloo et al., 2005; Kessler et al., 2006).

The spleen is the largest immune organ in mammals. The spleen is critical for the inhibition of systemic inflammation by vagal stimulation (Huston et al., 2006). The splenectomy abolished TNF reduction resulting from stimulation of vagal nerve efferents in sepsis (Huston et al., 2006). However, the vagus nerve does not affect splenic nerve activity (Bratton et al., 2012; Martelli et al., 2014a). Electrical stimulation delivered to the peripheral end of the cut vagus nerve did not drive action potentials in the splenic nerve (Bratton et al., 2012), which suggested that there is no disynaptic connection from the vagus to the spleen via the splenic nerves (Martelli et al., 2014a). The anti-inflammatory action of vagal stimulation may depend on the presence of noradrenaline-containing nerve terminals in the spleen (Martelli et al., 2014b). Splenic-denervation and catecholamine depletion abrogated the decrease in TNF level induced by activation of splenic sympathetic nerve in endotoxemia (Rosas-Ballina et al., 2008). Vida and his group (Vida et al., 2011) further found that the anti-inflammatory effect of vagal stimulation faded in β2 adrenergic receptor (β2AR) knockout mice, and β2AR agonist ameliorated inflammation in wild-type mice, but not in β2AR-deficient mice. Together with these components of the sympathetic nervous system, both sympathetic and parasympathetic systems could function synergistically to modulate inflammation.

ACh is the major vagal neurotransmitter. In addition to neurons, T lymphocytes also can synthesize non-neuronal source of Ach (Kawashima and Fujii, 2004; Rosas-Ballina et al., 2011). Except for the non-neuronal Ach from splenic T cells, blood ACh was mainly produced by circulating lymphocytes (Kawashima et al., 2015) and nicotine could elevate plasma Ach level (Kawashima et al., 1989). It is likely that these endogenous non-neuronal ACh may contribute to inhibition of the inflammatory cytokines together with nicotine by acting at α7nAChRs. The non-neuronal ACh is an important supplement to the intact cholinergic anti-inflammatory system.

Furthermore, we found that the heart injury and inflammation of the Vag + Nic mice was between those of the Vag group and the Nic group, and significantly lesser than that in the Vag group on day 7, indicating that nicotine administration can reverse the changes induced by vagotomy. The present study considered the α7nAChR subunit to be an essential part of vagal anti-inflammatory action. In 2003, Wang H et al. found that antisense oligonucleotides specific for the α7 subunit could suppress the TNF-inhibitory action of nicotine (Wang et al., 2003). Additionally, electrical stimulation of the vagus nerve could not inhibit the endotoxin-induced TNF increase in α7 subunit knockout mice, and the same stimulation of the vagus in wild-type mice significantly attenuated serum TNF levels (Wang et al., 2003; Pavlov and Tracey, 2004). In the present study, co-treatment with α7nAChR agonist nicotine decreased the level of proinflammatory cytokines, ameliorated myocardial lesions and improved depressed heart function in unilateral vagotomized mice infected with CVB3. After suppressing the cholinergic anti-inflammatory pathway by vagotomy, the chemical reactivation of this pathway by nicotine reduced the severity of viral myocarditis in mice. Therefore, we concluded that α7nAChR plays a key role in the cholinergic anti-inflammatory pathway, and this anti-inflammatory effect does not depend on the integrity of the vagus nerve. Vagotomy may reduce the release of acetylcholine from vagus nerve endings, with a decrease in the binding of α7nAChR, finally increasing pro-inflammatory cytokines to aggravate inflammation. Therefore, we supposed that a long-term reduction or absence of vagal tone may weaken the anti-inflammatory effect of the cholinergic anti-inflammatory pathway.

The present study demonstrated that changes of IL-1β, IL-6, and TNF-α were consistent at the mRNA level and protein level, suggesting that the cholinergic anti-inflammatory pathway is involved in the pre- and post-transcriptional processes of cytokine production. However, no significant differences were found in the mRNA and protein levels of IFN-γ among mice infected with CVB3. From this, we speculated that the cholinergic anti-inflammatory pathway did not affect the production and release of IFN-γ in viral myocarditis, the reduction in the inflammatory response was through the down-regulation of IL-1β, IL-6, and TNF-α, not IFN-γ. Leib et al. (2011) reported that the activation of cholinergic anti-inflammatory pathway in murine autoimmune myocarditis can inhibit the production of IL-6 and TNF-α in the spleen, but failed to affect IL-10, IL-13, IL-2, IL-17, and IFN-γ. After the activation of the cholinergic anti-inflammatory pathway, the release of other cytokines such as TNF-α, IL-1, IL-6, IL-18, and HMGB-1 was inhibited, but not the anti-inflammatory cytokine IL-10 (Wang et al., 2004). It was suggested that in different inflammatory diseases, the cytokines involved in cholinergic anti-inflammatory pathway may differ. Additionally, there is no significant change in the viral RNA abundance among the treated and untreated groups in the study. IFN-γ, secreted by T cells in acute viral myocarditis, is the essential cytokine for effective clearance of viral infections. CVB3 RNA was inversely correlated with the IFN-γ. IFN-γ deficiency resulted in increased viral replication in CVB3-induced myocarditis (Fairweather et al., 2005). Therefore, no significant changes in CVB3 RNA might be partly associated with the negative result in IFN-γ, and the cholinergic anti-inflammatory pathway might not affect CVB3 replication.

Several limitations should be noted in the present study. Firstly, the hemodynamic data was not measured in the study. There is evidence that heart rate was not altered 1 week after unilateral right cervical vagotomy, but blood pressure was decreased (Chen et al., 2008). Secondly, more selective α7nAChR agonists like AR-R17779 and GTS-21 (de Jonge and Ulloa, 2007) will support our result better.

## Conclusion

The findings indicate that vagal nerve plays an important role in mediating the anti-inflammatory effect in viral myocarditis, and that cholinergic stimulation with nicotine also plays its peripheral anti-inflammatory role and the effects of nicotine are independent of the integrity of vagal nerve in the model.

## Author Contributions

LY-C designed the experiment. LY-C, GL-S, CX-X, WL-P, ZD-P, LX-W, and LJ-F performed experiments. CX-X and GL-S contributed to analysis the experimental data. GL-S and CX-X wrote the paper.

## Conflict of Interest Statement

The authors declare that the research was conducted in the absence of any commercial or financial relationships that could be construed as a potential conflict of interest.
